# Clinical Decision-Making of Artificial Intelligence vs Medical Professionals in Patients With Syncope

**DOI:** 10.1016/j.jacadv.2025.102426

**Published:** 2025-12-19

**Authors:** Steven van Zanten, Thomas T. Boel, Jelle SY. de Jong, Babette Bais, Artur Fedorowski, Richard Sutton, Jasper L. Selder, Freek Giele, Christiaan Geertsma, Mike G. Scheffer, Joris R. de Groot, Frederik J. de Lange

**Affiliations:** a Reinier de Graaf Gasthuis, Department of Cardiology, Delft, the Netherlands; b Amsterdam University Medical Centre, University of Amsterdam, Heart Centre, Department of Clinical and Experimental Cardiology, Amsterdam Cardiovascular Sciences, Amsterdam, the Netherlands; c Reinier de Graaf Gasthuis, Reinier Academy, Delft, the Netherlands; d Department of Cardiology, Karolinska University Hospital, and Department of Medicine, Karolinska Institute, Stockholm, Sweden; e Department of Clinical Sciences, Lund University, Malmö, Sweden; f Department of Cardiology, National Heart and Lung Institute, Imperial College London, Hammersmith Hospital Campus, London, United Kingdom; g Rubicon B.V., Leusden, the Netherlands; h Amsterdam UMC, Location Vrije Universiteit Amsterdam, ICT Department, Amsterdam, the Netherlands

**Keywords:** artificial intelligence, clinical decision-making, diagnostic accuracy, education, general practitioner, referral letter, research, syncope

## Abstract

**Background:**

Artificial intelligence may improve diagnostic yield and accuracy in syncope.

**Objectives:**

The purpose of this study was to compare Generative Pretrained Transformer 4-Omni (GPT-4o) with medical professionals (MPs) in establishing syncope diagnoses and recommending interventions based on general practitioner’s referral letters to a syncope-unit.

**Methods:**

This three-phase study evaluated 55 anonymized referral letters. Phase-1: GPT-4o and MPs (12 physicians, 6 allied professionals) provided differential diagnoses. In Phase-2: all patients underwent 1.5 years of follow-up for recurrences and additional investigations. In Phase-3: a multidisciplinary committee established final diagnoses by adjudication. Diagnostic performance was assessed using a custom Diagnostic Precision Score (DPS), penalizing incorrect differential diagnoses from Phase-1. GPT-4o was tested in a privacy-safe environment and instructed with European Society of Cardiology guidelines.

**Results:**

Fifty-five letters were independently analyzed once by each of the eighteen MPs and by GPT-4o, yielding 1,045 assessments. Diagnostic yield, defined as any suggestion of a diagnosis, was 81.9% for physicians, 84.5% allied professionals, and 100% GPT-4o. Diagnostic performance, defined as the presence of the final diagnosis in the initial differential diagnosis, was 75.9% for GPT-4o, 48.6% and 36.7% for physicians and allied professionals. DPS was 22.9% for physicians (148.75/648), 12.6% for allied professionals (40.75/324), and −6.9% for GPT-4o (−4.00/54). GPT-4o incorrectly labeled 3 of 4 cardiac diagnoses as reflex syncope. GPT-4o, but not MPs, suggested additional lifestyle measures such as counterpressure maneuvers (29/55; 52.7%) and increased fluid intake (28/55; 50.9%).

**Conclusions:**

GPT-4o proposed a diagnosis in all cases; however, with a low DPS and is not yet suitable for unsupervised clinical use interpreting referral letters.

Syncope is a common medical emergency,^1^ yet its cause often remains undiagnosed.^2^ Dedicated syncope units (SUs) offer a guideline-based structured approach, increasing diagnostic yield up to 97%.^3^ The annual incidence of syncope is 1.91 per 1,000 person-years, with serious outcomes in 12.3%.^4^ As only a minority of patients seek medical care, the true prevalence is likely underestimated.^5^

General practitioners (GPs) play a key role in the diagnosis of syncope, as they are commonly the first point of contact. They refer patients to SUs for diagnosis or management.^6^ Implementing the European Society of Cardiology (ESC) syncope guidelines in primary care may improve diagnostic accuracy and reduce unnecessary referrals.

Artificial intelligence (AI), particularly machine and deep learning, is increasingly used to support diagnoses by improving consistency and aiding clinical judgment.^7^, ^8^, ^9^, ^10^, ^11^ Natural language processing with generative large language models (LLMs), such as Generative Pretrained Transformer 4-omni (GPT-4o),^12^ can analyze and generate clinical text^13^, ^14^, ^15^, ^16^ and have been explored in emergency care settings including syncope risk stratification.^17^ Such models may assist in triage and diagnosis, especially in complex cases like syncope, by extracting and interpreting information from electronic and unstructured records.^18^

GPT-4o^12^ is a multimodal language model trained on extensive textual data, including biomedical content. It does not reason like a clinician but identifies diagnostic patterns in narratives such as referral letters. LLMs may support judgment by improving consistency and reducing variability.^19^ Given the central role of history-taking in syncope diagnosis,^3^ LLMs aligned with ESC guidelines^2^ and supporting practical instructions^20^ may enhance diagnostic accuracy and reduce mismanagement.^21^ However, current application in syncope is limited due to lack of evidence and practical barriers.^16^^,^^22^ A better understanding of machine learning^16^^,^^23^ is essential to reduce unnecessary tests and admissions.^24^^,^^25^ LLMs may also reduce administrative burden and improve workflow.^26^ However, integration of LLMs with clinical practice still faces challenges regarding trust, legal frameworks, ethics, and data security.^23^^,^^27^^,^^28^

Traditional diagnostic tests, like history-taking, are highly operator dependent with interoperator variability, and limited by time and resources, resulting in wide practice variation.^29^ AI tools like GPT-4o may help standardize the approach.^10^ Although AI is not able to conduct a complete initial syncope evaluation as described,^2^ it may assist in data reduction and structure pathways.^2^^,^^3^^,^^6^ In this study, GPT-4o was specifically evaluated as a potential decision-support tool at the time of referral, aimed at supporting triage and early diagnostic orientation based on GP referral letters to the SU. We evaluated GPT-4o’s ability to derive a differential diagnosis. We compared the outcomes of GPT-4o with medical professionals (MPs), and with the final diagnosis based on a structured follow-up made by an expert committee, considered the gold standard for accurate diagnosis.^3^^,^^6^^,^^30^^,^^31^ To our knowledge, this is the first study evaluating clinical decision-making by analyzing the diagnostic performance of GPT-4o in real-world syncope assessment using GP referrals alone.

## Methods

### Patients

The Haaglanden SU database comprises patients referred to SUs at 3 hospitals between April 2015 and December 2022, with follow-up of 1.5 years. The present study represents an ancillary study of this prospective Haaglanden SU registry. For this study, we randomly selected a subset of patients referred from the GP between September 2019 and December 2022 who had follow-up and available referral letters. The referral letters of these patients were independently assessed by GPT-4o and a group of MPs, who were informed that the texts represented GP referral letters to the SU. The original content of the letters was not modified, but all patient identifiers were removed to ensure complete anonymization. We quantified the length of the GP referral letters used in this study by counting the total number of words in each document. In addition, we performed a focused word count restricted to the section describing the syncope episode (historical information) (Supplemental Figure 3).

**Figure 1:**
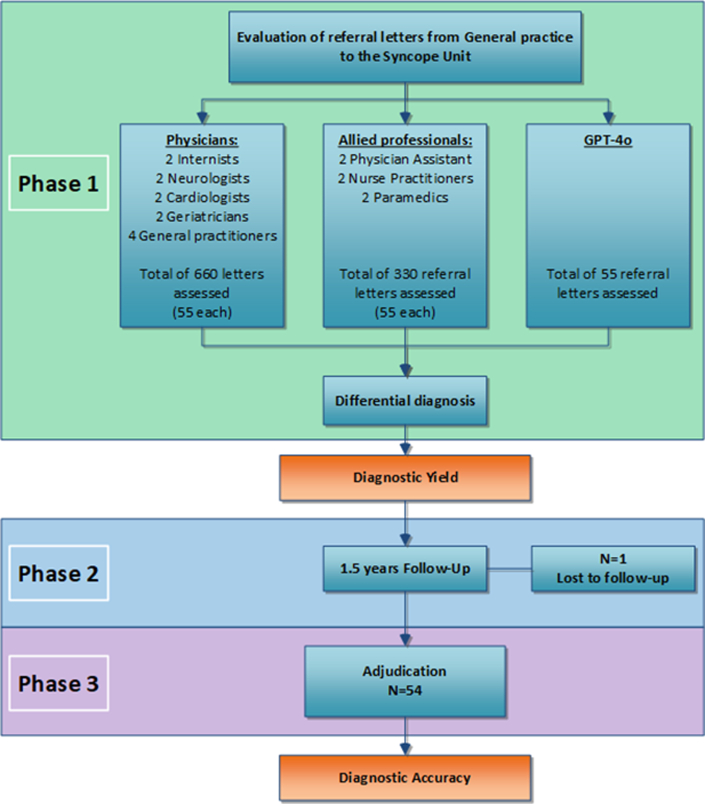
**Diagnostic Flowchart of GPT-4o and Medical Professionals** Phase-1: Independent analysis of 55 referral letters, each assessed once by GPT-4o (n = 55) and by eighteen medical professionals (n = 990), resulting in a total of 1,045 individual assessments. Phase-2: 1.5 years of critical follow-up. Phase-3: Diagnostic adjudication by an expert committee after 1.5 years of critical follow-up what leads to the final diagnosis (reference diagnosis). GPT = Generative Pretrained Transformer.

The group of MPs consisted of 12 physicians and 6 allied professionals. Physicians comprised 2 neurologists, 2 cardiologists, 2 geriatricians, 2 internists, and 4 GPs. Allied professionals comprised 2 nurse practitioners, 2 physician assistants, and 2 paramedics (Figure 1, Central Illustration). The study conforms to the Declaration of Helsinki, and the local Medical Ethics Review Committee approved the study (METC-nr: 18-061). All patients signed informed consent. Because this study introduced a novel evaluation step using GPT-4o, a separate protocol was required for ethical approval (REFSYNC-protocol, METC-nr: 2024.0124), specifically addressing data protection, General Data Protection Regulation compliance, and the requirement for human oversight. GPT-4o^12^ was tested in a controlled environment at the Amsterdam University Medical Centre to ensure patient safety and privacy.

**Central Illustration:**
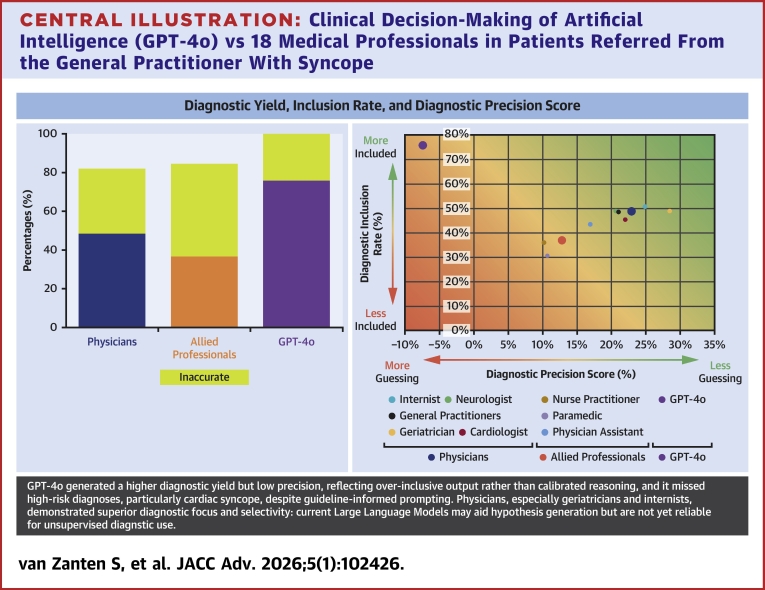
**Clinical Decision-Making of Artificial Intelligence (GPT-4o) vs 18 Medical Professionals in Patients Referred From the General Practitioner With Syncope** Diagnostic yield, inclusion rate, and precision by evaluator. Left: color-coded diagnostic yield for physicians, allied professionals, and GPT-4o. Right: inclusion rate and diagnostic precision score by group, stratified by specialty. Abbreviation as in Figure 1.

### Risk stratification

Risk stratification features of the ESC^2^ syncope guidelines (Supplemental Figure 1) were used to structure the initial syncope evaluation of the referral letter. We systematically assessed whether these features (low risk, high minor, and high major) were included in the referral letter. As these risk categories are based on information from history, physical examination, and electrocardiogram.

### Phases of diagnosis

Phase-1: Diagnostic Yield: Based on the information available in the referral letter, MPs and GPT-4o were instructed to establish a diagnosis, either one or a differential diagnosis (Phase-1) (Figure 1).^3^^,^^6^^,^^30^^,^^32^ They were explicitly instructed to include all plausible causes, including alternative diagnoses. Diagnostic yield was defined as the proportion of patients in whom a diagnosis was established.^30^ To facilitate GPT-4o and MPs, we provided historical clues from the ESC guidelines to diagnose syncope (Supplemental Figure 2).^2^

Phase-2: Critical follow-up: During 18-month follow-up, all information on syncope recurrences, additional testing performed, any admissions or consultation for syncope, and whether the diagnosis after the initial visit in the SU was changed or not, was collected through 2 questionnaires (Phase-2) (Figure 1). The final questionnaire was sent after 18 months.

Phase-3: Final diagnosis: After follow-up, using all data collected, diagnoses were adjudicated by an independent committee (F.d.L., J.d.J., T.B.) to determine the reference diagnosis: hereinafter referred to as the final diagnosis (Phase-3) (Figure 1). Prior to the consensus meeting, each committee member independently reviewed the data of every case and established a diagnosis. When there was unanimous agreement, or when 2 members agreed and a third made no diagnosis, a final diagnosis was established. In case of disagreement, the case was discussed in a face-to-face consensus meeting. If this discussion resulted in consensus, a final diagnosis was established. The case was deemed unexplained if no consensus was reached^3^^,^^31^^,^^33^^,^^34^ (Phase-3) (Figure 1).

### Diagnostic inclusion rate and precision

**Figure 2:**
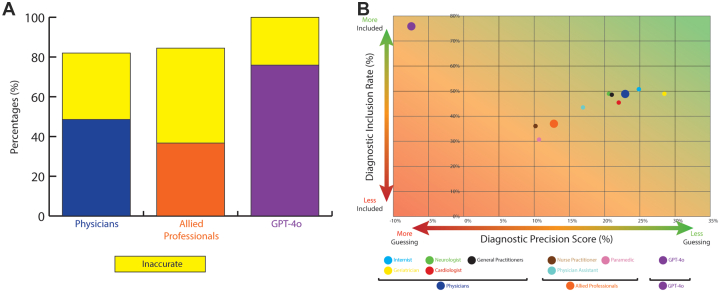
**Diagnostic Yield and Diagnostic Precision Score** (A) Combined colored and yellow bars indicate total diagnostic yield. Colored bars reflect diagnostic inclusion rate (ie, proportion of final diagnoses that was included in the list of differential diagnoses of Phase-1); yellow bars show inaccurate differential diagnosis lists (ie, the final diagnosis was not included). (B) The y-axis shows diagnostic inclusion rate (%); the x-axis reflects Diagnostic Precision Score (%). The background gradient integrates both dimensions: green indicates concise and accurate reasoning; red reflects diffuse and inaccurate, and imprecise. Abbreviation as in Figure 1.

We first assessed diagnostic accuracy which was assessed by the diagnostic inclusion rate which was defined as the proportion of final diagnoses (Phase-3) included in the list of differential diagnoses of Phase-1. This analysis was conducted across 3 groups and subgroups: physicians, allied professionals, and GPT-4o (Figure 2A).

Secondly, we assessed the Diagnostic Precision Score (DPS). We calculated the DPS by weighing the included final diagnosis against the number of diagnoses in the differential diagnosis from Phase-1. We rewarded diagnostic inclusion rate for each case, while penalizing diagnostic over-inclusiveness by a penalty-score. More specifically: +1 point was awarded if the final diagnosis from Phase-3 was included in the list of the differential diagnosis from Phase-1. When multiple diagnoses were listed in Phase-1, a fixed penalty-score was applied to each incorrect diagnosis and was subtracted from +1 point if the final diagnosis was included, and from 0 if not mentioned. This penalty-score was calculated as 1 divided by the largest possible number of incorrect differential diagnoses in the entire cohort. The DPS was reported by each subgroup (GPT-4o n = 1, physicians n = 12, allied professionals n = 6) as an average per assessed referral letter. For example, see Supplemental Table S1.

The DPS system was based on Bayesian reasoning^35^^,^^36^ and corrects for confidence in the correct diagnosis. Although not based on a formal statistical model, this penalty acts as a simple way to reflect how diagnostic certainty is diluted when too many incorrect options are included in the differential diagnoses. It offers a structured, interpretable framework for evaluating differences in diagnostic decision-making in both MPs and AI-based systems.

### Diagnostic safety

Diagnostic safety is defined as recognizing a cardiac cause of syncope. Diagnostic safety was quantified through 2 different strategies. First, we defined diagnostic safety by determining the proportion of cardiac syncope diagnoses confirmed by GPT-4o and MPs in Phase-1 compared to the final cardiac diagnosis of Phase-3. For GPT-4o, only the first proposed diagnosis was considered, and for MPs, only single, explicitly cardiac diagnoses were included; cases with multiple diagnoses were excluded. Secondly, vice versa, we compared all patients with a final cardiac syncope diagnosis of Phase-3 to the initial Phase-1 diagnosis of GPT-4o and MPs.

### Training artificial intelligence

A tailored system prompt was developed using the RICCE framework (Role, Instructions, Context, Constraints, Examples)^37^ to optimize the performance of GPT-4o in syncope evaluation. It consisted of:

1. Structured RICCE guidance for diagnostic reasoning
2. Text extracts from the 2018 ESC syncope guidelines^2^ and practical instructions^20^ to improve clarity and reduce noise by stripping of nonessential content but retaining tables and legends as plain text
3. A case-based teaching strategy, iteratively refined using real-world examples.

Due to the model’s text-only nature, visual elements were excluded. The final prompt, >90k tokens, was deployed as a single system prompt using GPT-4o's extended 128k token context window. GPT-4o (version 2024-05-13) was instructed to produce structured five-step outputs for each case in line with the guidelines:^2^^,^^20^

1. Clarifying questions based on the GP referral letter
2. Diagnosis with rationale and differential diagnosis
3. Diagnostic certainty
4. Recommendations for the GP
5. Hospital referral advice for the syncope specialist

This tailored approach was designed to reduce documentation errors, improve consistency, and support clinical decision-making in the evaluation and management of syncope. It was extensively tested using a series of clinical cases meeting predefined historical clues, as previously described. Multiple runs were performed per case, iteratively refining the prompt until outcomes aligned with ESC guideline classifications. After internal validation, referral letters (distinct from those used in this article) were uploaded for final verification. Upon consensus, the system was finalized for production.

### Statistical analysis

Continuous variables are presented as mean ± SD or median with 25th-75th percentiles (Q1-Q3) while categorical data are reported as counts and percentages. A logistic regression analysis was conducted to evaluate the association between the number of clarifying questions posed by GPT-4o and the likelihood of establishing the correct diagnosis. The number of questions served as the independent variable, and the correct final diagnosis functioned as the dependent variable. ORs with corresponding 95% CIs and *P* values are reported. Given the skewed distribution of data, percentages were used to facilitate interpretation.

Inclusion rates were determined and expressed as proportions with 95% CIs, providing an estimate of the inclusion rates and its statistical uncertainty.

To compare diagnostic performance across assessor groups, a one-way analysis of variance was performed using the DPS per case as the dependent variable. For the clinician groups, the per-case was calculated as the mean across eighteen MPs from different specialties, and this mean value was compared with the per-case DPS generated by GPT-4o. Owing to unequal group sizes and nonhomogeneous variances (as assessed by Levene’s test), post hoc comparisons were conducted using the Games–Howell procedure. This test accounts for heteroscedasticity and adjusts for multiple comparisons. Results are presented as mean differences with corresponding 95% CIs and *P* values. A *P* value <0.05 was deemed significant. Analyses were performed using International Business Machines (IBM) Statistical Package for the Social Sciences (SPSS) Statistics, version 25 (International Business Machines Corporation).

## Results

A sample of 55 patients was randomly selected from the overall cohort. These 55 referral letters were assessed by MPs (n = 18) and GPT-4o in total 1,045 times (19x55). Clinical characteristics of the included patients upon referral are summarized in Table 1.

**Table 1:** Clinical Characteristics (N = 55)

Age (y)	
Mean (SD)	61 (17)
Median (Q1, Q3)	63 (50, 75)
Min, max	21-87
Female	30 (54.5%)
Height (centimeters)	
Mean (SD)	173 (9)
Median (Q1, Q3)	172 (167, 180)
Min, max	155-193
Weight (kilogram)	
Mean (SD)	77 (13)
Median (Q1, Q3)	75 (66, 87)
Min, max	54-105
Body mass index (kg/m^2^)	
Mean (SD)	26 (3.7)
Median (Q1, Q3)	25 (23, 28)
Min, max	18-36

### Referral letter

Prior to referral, 30.9% (17/55) of patients were using antihypertensive medication, of which 29.4% (5/17) were on single regimen and 705% (12/17) used more than one antihypertensive medication (Table 2). None of the letters from GPs reported reviewing or adjusting medication prior to referral. High-risk syncope (high-major or one high-minor with structural heart disease) was identified in 25.4% (14/55) of cases. Only one high-minor (Supplemental Figure 1) in the absence of structural heart disease was found in 41.8% (23/55) (Table 3).^2^ The referral letters contained a mean of 350 words (SD: 217, range: 58-1,158), whereas the syncope-related clinical history information averaged 150 words (SD: 171, range: 2-1,086).

**Table 2:** Medication and Diagnostic Tests (N = 17)

Calcium antagonist	8 (47.0%)
Angiotensin-converting enzyme inhibitors	7 (41.1%)
Beta-blockers	7 (41.1%)
Diuretics	6 (35.2%)
Angiotensin receptor blockers	4 (23.5%)
Nitrates	2 (11.7%)
Alpha-blockers	1 (5.8%)
**Use of antihypertensive medication(s) (n = 17)**	
Use of 1	5 (29.4%)
Use of 2	7 (41.1%)
Use of 3	4 (23.5%)
Use of 4	1 (5.8%)
**Diagnostic test performed before referral (n = 55)**	
Physical examination	45 (81.8%)
Blood pressure measurement	41 (74.5%)
Heart frequency measurement	36 (65.4%)
12-lead electrocardiogram	17 (30.9%)
Laboratory test	7 (12.7%)
Supine and standing blood pressure measurement	2 (3.6%)
Holter monitoring	2 (3.6%)
24-h blood pressure measurement	1 (1.8%

**Table 3:** Risk Stratification Syncopal Event

Not mentioned	10
Low risk	22
High risk (minor)	23
High risk (major)	4
Past medical history	
Low risk	52
High risk (major)	3
Physical examination	
Not mentioned	10
Low risk	42
High risk (major)	3
Electrocardiogram	
Not mentioned	38
Low risk	13
High risk (minor)	0
High risk (major)	4

Outcome of the risk stratification following the ESC guideline on syncope. Risk stratification features reported in 55 GP referral letters. Each letter refers to a single index episode unless multiple episodes described in the letters and may exceed 55 when multiple episodes were mentioned.

### Phases of diagnosis

Phase-1: The diagnostic yield, defined as establishing at least one diagnosis, was 81.9% (541/660; average 45 ± 4.7) for physicians, 84.5% (279/330; average 45 ± 4.4) for allied professionals, and 100% (55/55) for GPT-4o (Figure 2A).

Phase-2: Critical follow-up was performed in all patients. However, one patient was lost to follow-up (Figure 1). Therefore, the assessment was confined to 54 cases. Assessment of the diagnostic inclusion rate and DPS was performed in 54 cases for GPT-4o, 648 (12×54) for physicians, and 324 (6×54) for allied professionals, respectively.

Phase-3: The adjudicated final diagnosis of Phase-3, established by the expert committee, was reflex syncope in 46.2% (25/54) of cases, unexplained syncope in 24.0% (13/54), orthostatic hypotension in 20.3% (11/54), cardiac syncope in 7.4% (4/54), and psychogenic pseudosyncope in 1.8% (1/54). Of the 4 patients with cardiac syncope, 3 were caused by atrial fibrillation, and one patient showed ischemic ST-segment deviations during diagnostic evaluation, leading to the detection of myocardial ischemia and subsequent referral for coronary artery bypass grafting.

### Diagnostic inclusion rate, safety, and precision

With one case lost to follow-up, GPT-4o correctly proposed the adjudicated final diagnosis in Phase-1 in 41/54 cases which makes the diagnostic inclusion rate of 75.9% (95% CI: 64.5-87.3), compared with 48.6% for the physicians (95% CI: 44.8-52.4; 315/648 letters) and 36.7% for the allied professionals (95% CI: 31.5-41.9; 119/324 letters) (Figure 2A).

Within the subgroup of physicians, internists included the adjudicated final diagnosis in their initial differential diagnosis in 50.9% of cases (95% CI: 41.5-60.3; 55/108), followed by neurologists in 49.0% (95% CI: 39.6-58.4; 53/108), geriatricians in 49.0% (95% CI: 39.6-58.4; 53/108), GPs in 48.6% (95% CI: 41.9-55.3; 105/216), and cardiologists in 45.3% (95% CI: 35.9-54.7; 49/108) (Figure 2B, X-axis).

Within the allied professionals subgroup, physician assistants included the adjudicated final diagnosis in the differential diagnosis in 43.5% of cases (95% CI: 34.1-52.9; 47/108), nurse practitioners in 36.1% (95% CI: 27.0-45.2; 39/108), and paramedics in 30.5% (95% CI: 21.8-39.2; 33/108) (Figure 2B, X-axis).

More than one diagnosis was suggested in Phase-1 by GPT-4o in 87.3% (48/55) of cases (mean = 4.02 ± 0.77 diagnoses), whereas this proportion was 40.3% (266/660) for the physicians (mean = 1.49 ± 0.72 diagnoses) and 29.1% (96/330) for the allied professionals (mean = 1.31 ± 0.58 diagnoses).

The maximum number of diagnoses provided per case by both the MPs and GPT-4o was 5. Consequently, the penalty-score for each incorrect diagnosis in the list of differential diagnosis was 0.25 (see Methods). With this we calculated the DPS for every case, as described above. Overall DPS was 22.9% in the physician group, and 12.6% in the allied professional group. GPT-4o yielded a DPS of −6.9% (Figure 2B, y-axis).

Within the subgroup of physicians, the geriatricians showed a DPS of 28.5%, internists 24.8%, cardiologists 22.0%, GPs 20.8%, and neurologists 20.6% (Figure 2B, y-axis). Among the allied professionals, physician assistants scored a DPS of 16.9%, paramedics 10.6%, and nurse practitioners 10.2% (Figure 2B, y-axis).

Physicians achieved a significantly higher DPS compared to both allied professionals (mean difference 0.103; 95% CI: 0.008-0.199; *P* = 0.030) and GPT-4o (mean difference 0.303; 95% CI: 0.107-0.500; *P* < 0.001). The difference between allied professionals and GPT-4o was not significant (mean difference 0.200; 95% CI: −0.003 to 0.403; *P* = 0.055).

Internists reached a significantly higher DPS compared with GPT-4o (mean difference 0.322; 95% CI: 0.012-0.632; *P* = 0.036), as also did geriatricians (mean difference 0.359; 95% CI: 0.050-0.668; *P* = 0.011). All other pairwise comparisons were not statistically significant.

**Figure 3:**
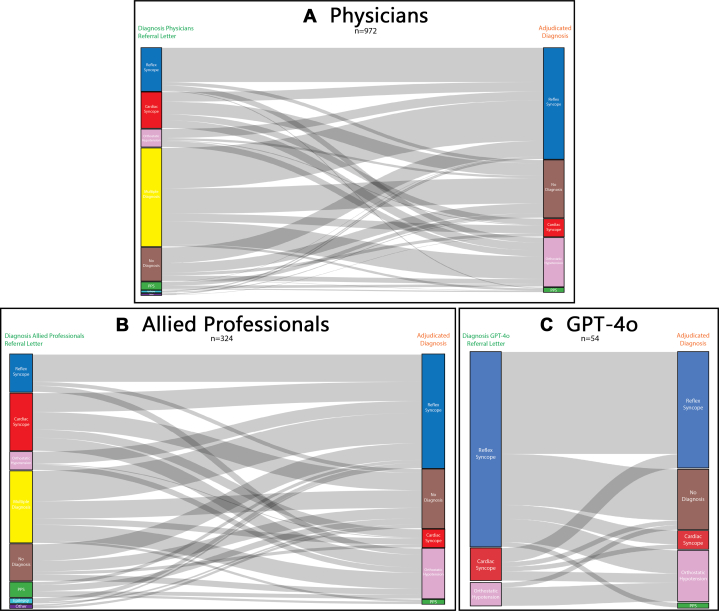
**Diagnostic Flowchart of Phase-1 Diagnosis Compared to the Phase-3 Final Diagnosis for the Physicians, Allied Professionals, and GPT-4o** The diagnostic classification of syncope cases based on 54 referral letters (one was lost to follow-up), of the initial diagnoses (Phase-1) compared to the final diagnosis (Phase-3) (right side). This was done for physicians (A), allied professionals (B), and GPT-4o (C). PPS = psychogenic pseudosyncope; other abbreviation as in Figure 1.

Firstly, to quantify diagnostic safety, GPT-4o assigned a cardiac diagnosis in 7 of 54 referral letters (12.9%), of which one was in line with the final diagnosis, and 6 had a final diagnosis of reflex syncope (n = 3), orthostatic hypotension (n = 2), or no diagnosis (n = 1). Allied professionals established a diagnosis of cardiac syncope in 23.4% of referral letters (76/324; average 13 ± 3), of which eleven were in line with the final diagnosis, and 65 had a final diagnosis of reflex syncope (n = 25), orthostatic hypotension (n = 17), and no diagnosis (n = 23). Physicians established a cardiac diagnosis in 14.9% of referral letters (97/648; average 8 ± 4), of which sixteen were in line with the final diagnosis, and 81 had a final diagnosis of reflex syncope (n = 26), orthostatic hypotension (n = 21), and no diagnosis (n = 34). See Figures 3A to 3C for specifications of diagnostic safety outcomes.

Secondly, we looked if the final diagnosis was correctly identified by the reviewers of the expert committee. 4 of 54 patients had a final diagnosis of cardiac syncope. Of those 4 cases, GPT-4o made the diagnosis reflex in 3, and cardiac in one. Allied professionals proposed diagnosis for a total of 24 cardiac cases of which eleven (45.8%) were classified as cardiac, 8 (33.3%) within multiple diagnoses, 4 (16.6%) received no diagnosis, and one (4.1%) was diagnosed with orthostatic hypotension. Physicians proposed diagnosis for 48 cardiac cases, of which 21 (43.7%) with multiple diagnoses, sixteen (33.3%) classified as cardiac, 8 (16.6%) with no diagnosis, 2 (4.1%) as orthostatic hypotension, and one (2.0%) as other diagnosis.

### Suggested additional diagnostic tests, therapeutic interventions, and additional questions

**Figure 4:**
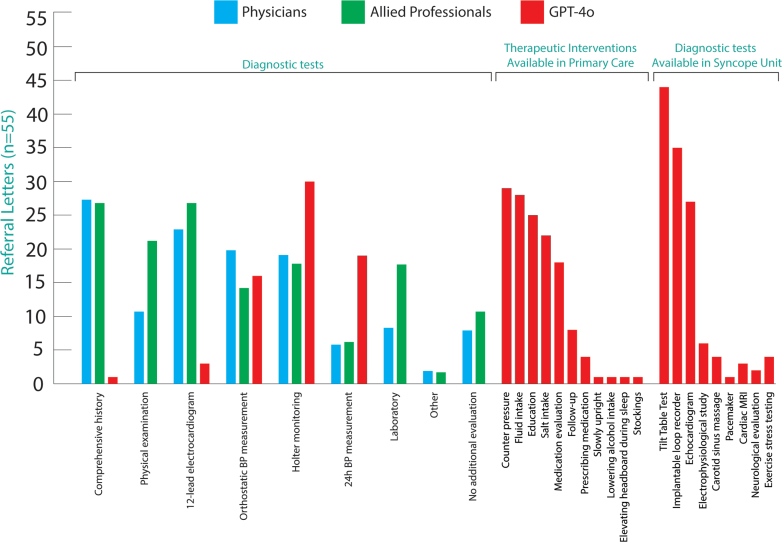
**Suggestions for Diagnostic Tests and Therapeutic Interventions After Phase-1** Suggested therapeutic and diagnostic interventions from the eighteen medical professionals and GPT-4o categorized for the final diagnosis. The total amount of evaluated referral letters is 19x55 = 1,045. Overall means are presented separately for physicians, allied professionals, and GPT-4o. BP = blood pressure; MRI = magnetic resonance imaging; other abbreviation as in Figure 1.

The most frequently proposed diagnostic tests, predominantly suggested by MPs, that could be performed prior to referral, were history-taking (49.0%), 12-lead ECG (44%), and Holter monitoring (34.5%). GPT-4o most often recommended tests requiring a SU, including tilt-table testing (80.0%), implantable loop recorder (63.6%), and Holter monitoring (54.5%). Therapeutic interventions were suggested by GPT-4o but not by MPs, predominantly counterpressure maneuvers (52.7%), increased oral fluid intake (50.9%), and patient education (45,4%). For a complete overview of all proposed investigations and interventions, see Figure 4.

GPT-4o suggested additional questions based on the content of the referral letters. The number of questions asked was not significantly associated with diagnostic certainty (OR: 0.789; 95% CI: 0.548-1.137; *P* = 0.20). The group of MPs did not suggest additional questions.

When the diagnosis of the MP was accurate, significantly fewer tests were suggested (OR: 1.157; 95% CI: 1.067-1.255; *P* < 0.001). An accurate diagnosis by GPT-4o was not associated with the suggestion for fewer tests (OR: 0.726; 95% CI: 0.380-1.387; *P* = 0.33).

## Discussion

This study for the first time assessed the clinical decision-making of AI by comparing the diagnostic performance of GPT-4o, with that of MPs for the interpretation of syncope data provided in referral letters from the GPs to the SU, building on previous theoretical work on machine learning in emergency care.^17^

### Risk stratification

The ESC syncope guideline recommends structured risk stratification based on clinical history, comorbidities, physical examination (including supine and standing blood pressure), and ECG.^2^ Such information in referral letters of the GP can be valuable for diagnosis. However, documentation was highly variable: while some letters supported a low- or high-risk classification, many lacked sufficient detail to apply the ESC framework effectively (Table 3). Risk assessment may have been performed but was generally not clearly documented.

When examining the word counts of the GP referral letters, we observed substantial variability in both the overall length and the level of clinical detail provided. The finding that the syncope-related content represents less than half of the total word count highlights the importance of accounting for information density when evaluating AI performance in this setting. This variability also underscores that referral practices may differ considerably across health care systems, which should be considered when generalizing our findings. Longer and more detailed letters may enable more accurate GPT-4o output, whereas very brief letters may limit its performance.

### Diagnostic yield, inclusion rate, precision, and safety

GPT-4o proposed a differential diagnosis in all cases. This reflects GPT-4o's apparent tendency always to provide an answer in any circumstance. GPT-4o never indicates diagnostic uncertainty or acknowledges when no diagnosis is appropriate. From a diagnostic point of view, this behavior inflates diagnostic yield and does so by overconfident pattern recognition rather than clinical judgment. Notably, GPT-4o frequently included a cardiac diagnosis in its differential diagnosis, regardless of context, and regardless of clues that would direct clinical reasoning. This over inclusiveness contributed to a relatively high diagnostic yield but was largely driven by diagnostic gambling (Figure 2).

MPs also often adopted a cautious approach, withholding diagnosis in uncertain cases, resulting in a diagnostic yield of 82% and 85% for physicians and allied professionals, respectively.

GPT-4o outperformed MPs in overall inclusion rate (75.9%) but missed 3/4 cardiac causes of syncope (Figure 3), consistent with earlier findings showing high AI accuracy (up to 85.0%) in detecting syncope.^38^^,^^39^ GPT-4o was unable to express diagnostic uncertainty, even when withholding a diagnosis was appropriate.

Both MPs and GPT-4o still missed substantial proportions of cardiac cases (25.0% and 33.3%, respectively). Cardiac syncope was most frequently listed as a primary or one of multiple diagnoses but was sometimes misclassified as no diagnosis, reflex syncope, or orthostatic hypotension. These errors may stem from insufficient clinical information in referral letters.

GPT-4o’s negative DPS (−6.9) illustrates that the model was insufficiently able to discriminate relevant from irrelevant diagnoses. GPT-4o frequently provided long lists of diagnoses (up to 5 per case). This is characteristic of LLMs, which optimize plausibility rather than prioritization.^40^^,^^41^ Physicians outperformed both GPT-4o and allied professionals in DPS (*P* < 0.001 and *P* = 0.03, respectively), with geriatricians (*P* = 0.01) and internists (*P* = 0.03) achieving the highest scores. Compared to LLM, clinical experience likely helped physicians to focus on what really mattered. These findings align with studies highlighting the selective efficiency of experienced clinicians.^42^ To the best of our knowledge, this type of negatively weighted differential scoring has not been previously applied in comparative diagnostic studies.

Looking at diagnostic safety, GPT-4o missed 75.0% of cardiac syncope diagnoses and misclassified these as reflex syncope. Allied professionals and physicians were more accurate (45.8% and 33.3%), likely reflecting the value of clinical reasoning in high-risk conditions. Our finding of false reassurance by GPT-4o in high-risk syncope cases supports concerns about safety of applying AI in decision-making without expert oversight.

### Performance limitations and clinical caution

Generative LLMs like GPT-4o are not designed for clinical reasoning. They operate through predictive text completion rather than rule-based deduction.^37^ As shown here, GPT-4o was insufficiently able to differentiate between benign and high-risk syncope causes and missed key cardiac cases.^39^ Additionally, GPT-4o frequently made overconfident misclassifications. This is consistent with concerns raised in prior reviews of machine learning in syncope, which noted limitations such as lack of external validation, limited generalizability, and insufficient diagnostic standards.^16^^,^^17^

Systematic issues include hallucinations, miscalibrated certainty, and poor recognition of red flags, reinforcing their unsuitability for autonomous clinical use.^38^, ^39^, ^40^ Structured input and model refinement with adjudicated gold standard diagnoses are useful to train AI and are essential before clinical integration in syncope care.

GPT-4o may thus provide practical support for GPs in deciding on referral and next steps, and for nonsyncope specialists in planning initial hospital evaluation, particularly through its structured therapeutic suggestions.

### Learning opportunities from artificial intelligence and clinician approaches

Despite GPT-4o’s limitations, LLMs offer advantages in syncope assessment. This study illustrates the complementary roles of AI and MPs. GPT-4o’s exhaustive therapeutic suggestions (Figure 4) may inform structured, hypothesis-free treatment pathways, while MPs nuanced restraint anchors safety-oriented decision-making. More specifically, given the absence of national syncope guidelines for the GPs in the Netherlands, these findings may underscore the need for structured diagnostic syncope pathways in primary care. Such an adapted pathway may explicitly incorporate therapeutic interventions in cases where cardiac syncope is unlikely to ensure appropriate management and thereby may avoid unnecessary patient referral to secondary care.

### Strengths and weaknesses

This study has 3 strengths: firstly, it provides direct comparison between AI and MPs. Secondly, AI was trained according to a widely followed guideline and, thirdly, it included outcome-based diagnostic adjudication. The study’s main weakness is the small numbers of cases included. It is anticipated that this will be corrected in the future.

## Conclusions

In this comparison of GPT-4o and MPs assessing syncope referral letters, GPT-4o achieved a higher diagnostic yield with a low diagnostic precision, reflecting over inclusive output rather than calibrated reasoning. Despite guideline-informed prompting, GPT-4o missed high-risk diagnoses, particularly cardiac syncope. Physicians, especially geriatricians and internists, demonstrated far better diagnostic focus and selectivity. While promising for generating a hypothesis, current LLMs lack reliability for unsupervised diagnostic use.

Perspectives **COMPETENCY IN MEDICAL KNOWLEDGE:** AI tools like GPT-4o show promising results in identifying reflex syncope, clinical expertise remains essential for recognizing high-risk conditions such as cardiac syncope.

**TRANSLATIONAL OUTLOOK:** Future efforts should focus on integrating AI support into structured triage frameworks. Improved training data and better referral letter quality could enhance AI performance, but currently expert oversight remains critical to ensure safe and accurate syncope management.

## Funding support and author disclosures

The authors have reported that they have no relationships relevant to the contents of this paper to disclose.
