# Supplementary material for: Clinical Decision-Making of Artificial Intelligence vs Medical Professionals in Patients With Syncope
Source: JACC Adv. 2025 Dec 19;5(1):102426. doi: 10.1016/j.jacadv.2025.102426 (PMC12794501; doi:10.1016/j.jacadv.2025.102426)
Supplement: Supplemental_Material [file mmc1.docx]

**Supplementary figure 1 (Obtained from the 2018 ESC syncope Guideline)**

| **SYNCOPAL EVENT** | |
| --- | --- |
| Low-risk | |
| Associated with prodrome typical of reflex syncope (e.g. light-headedness, feeling of warmth, sweating, nausea, vomiting) | |
| After sudden unexpected unpleasant sight, sound, smell, or pain | |
| After prolonged standing or crowded, hot places | |
| During a meal or postprandial | |
| Triggered by cough, defaecation, or micturition | |
| With head rotation or pressure on carotid sinus (e.g. tumour, shaving, tight collars) | |
| Standing from supine/sitting position | |
| High-risk | |
| **Major** | |
| New onset of chest discomfort, breathlessness, abdominal pain, or headache | |
| Syncope during exertion or when supine | |
| Sudden onset palpitation immediately followed by syncope | |
| **Minor** (high-risk only if associated with structural heart disease or abnormal ECG): | |
| No warning symptoms or short (<10 s) prodrome | |
| Family history of SCD at young age | |
| Syncope in the sitting position | |
| **PAST MEDICAL HISTORY** | |
| Low-risk | |
| Long history (years) of recurrent syncope with low-risk features with the same characteristics of the current episode | |
| Absence of structural heart disease | |
| High-risk | |
| **Major** | |
| Severe structural or coronary artery disease (heart failure, low LVEF or previous myocardial infarction) | |
| **PHYSICAL EXAMINATION** | |
| Low-risk | |
| Normal examination | |
| High-risk | |
| **Major** | |
| Unexplained systolic BP in the ED <90 mmHg | |
| Suggestion of gastrointestinal bleed on rectal examination | |
| Persistent bradycardia (<40 b.p.m.) in awake state and in absence of physical training | |
| Undiagnosed systolic murmur | |
| **ECGa** | |
| Low-risk | |
| Normal ECG | |
| High-risk | |
| **Major** | **Minor** (high-risk only if history consistent with arrhythmic syncope) |
| • ECG changes consistent with acute ischaemia  • Mobitz II second- and third-degree AV block  • Slow AF (<40 b.p.m.)  • Persistent sinus bradycardia (<40 b.p.m.), or repetitive sinoatrial block or sinus pauses >3 seconds in awake state and in absence of physical training  • Bundle branch block, intraventricular conduction disturbance, ventricular hypertrophy, or Q waves consistent with ischaemic heart disease or cardiomyopathy  • Sustained and non-sustained VT  • Dysfunction of an implantable cardiac device (pacemaker or ICD)  • Type 1 Brugada pattern  • ST-segment elevation with type 1 morphology in leads V1–V3 (Brugada pattern)  • QTc >460 ms in repeated 12-lead ECGs indicating LQTS | • Mobitz I second-degree AV block and 1°degree AV block with markedly prolonged PR interval  • Asymptomatic inappropriate mild sinus bradycardia (40–50 b.p.m.), or slow AF (40–50 b.p.m.)  • Paroxysmal SVT or atrial fibrillation  • Pre-excited QRS complex  • Short QTc interval (≤340 ms)  • Atypical Brugada patterns  • Negative T waves in right precordial leads, epsilon waves suggestive of ARVC |
|  |  |
|  |  |
|  |  |
|  |  |
|  |  |
|  |  |
|  |  |
|  |  |
|  |  |
| AF = atrial fibrillation; ARVC = arrhythmogenic right ventricular cardiomyopathy; AV = atrioventricular; BP = blood pressure; b.p.m. = beats per minute; ECG = electrocardio gram; ED = emergency department; ICD = implantable cardioverter defibrillator; LQTS = long QT syndrome; LVEF = left ventricular ejection fraction; SCD = sudden cardiac  death; SVT = supraventricular tachycardia; VT = ventricular tachycardia.  a Some ECG criteria are per se diagnostic of the cause of the syncope (see recommendations: Diagnostic criteria); in such circumstances appropriate therapy is indicated without  further investigations. We strongly suggest the use of standardized criteria to identify ECG abnormalities with the aim of precise diagnosis of ECG-defined cardiac syndromes in  ED practice. | |

**Supplementary Figure 2 (Obtained from the 2018 ESC Syncope Guideline Web Practical Instructions)**

| **Checklists of historical clues to diagnose transient loss of consciousness** | | |
| --- | --- | --- |
| **Historical clue** | **Possible diagnosis** | **References, comments, definition** |
| Supine position (awake) | • Cardioinhibitory VVS through pain or fear • Arrhythmia • PPS and PNES | Epilepsy Cardiac |
| During normal sleep | • Epilepsy • Arrhythmia • If prodrome of VVS causing awakening + syncope thereafter: “sleep syncope” | Epilepsy Sleep syncope |
| Sitting | • All causes (including “orthostatic VVS” and classical OH) | For VVS |
| Standing for some period | • All causes • If TLOC occurs only while standing: OH, orthostatic VVS |  |
| Couple of steps after standing up or straightening from bending or squatting position | Initial OH and classical OH | Initial OH |
| Micturition, defaecation | Situational reflex syncope (note: defaecation and diarrhoea may act as triggers for VVS but also as symptoms of VVS) | Micturition Defaecation |
| Coughing | Situational syncope (usually prolonged intensive coughing, often in smokers with lung disease) |  |
| Swallowing | Situational syncope (usually oesophageal disease) |  |
| Laughing out loud, telling jokes, unexpectedly meeting an acquaintance | Cataplexy (ask about excessive daytime sleepiness) |  |
| Laughter | Situational reflex syncope (very rare) |  |
| During and after eating | • All causes (a specific circumstance) – Only during/after eating (15 minutes): postprandial hypotension, particularly in the elderly and with autonomic failure- If preferentially during meals: arrhythmia/Brugada syndrome | Postprandial hypotension |
| Head movements, pressure on the neck, shaving | Spontaneous type of carotid sinus syncope |  |
| Ear pain, instrumentation | Classical VVS |  |
| During physical exercise | • Cardiac structural- Cardiac arrhythmic: AV block, LQTS1, catecholaminergic VT - May occur in autonomic failure- VVS in very young/teenagers | Effort |
| Directly after cessation of physical exercise | • Post-exercise hypotension in middle-aged and elderly people: autonomic failure • Young people: VVS, particularly in trained athletes | AF  VVS |
| During arm exercise | Steal syndrome (very rare) |  |
| Palpitations | • Cardiac: tachyarrhythmia • Postural tachycardia in VVS, POTS | Syncope vs. seizure |
| Strong emotions other than fear (e.g. argument) | • Cataplexy • Arrhythmia: catecholaminergic polymorphic VT; also during exercise, in children and young adults | Cataplexy Catecholaminergic polymorphic VT |
| Startling (e.g. alarm clock) | • LQTS2 • Startle epilepsy | LQTS2 Epilepsy |
| During fever | • VVS (more often) • Brugada syndrome | Many case reports but no systematic counts on syncope with/without fever |
| Flashing lights | Epilepsy with photosensitivity |  |
| Sleep deprivation | • Epilepsy• VVS | Epilepsy |
| Heat/warmth/hot bath | • VVS • Classical OH | VVS OH Syncope vs. seizure |
| AF=atrial fibrillation; AV=atrioventricular; LQTS=longQTsyndrome; OH=orthostatichypotension; PNES=psychogenic non-epileptic seizures; POTS=postural orthostatic tachycardia syndrome; PPS=psychogenic pseudosyncope; TLOC=transient loss of consciousness; VT=ventricular tachycardia; VVS=vasovagal syncope. | | |

**Supplementary Figure 3**

**Referral letter for a combined appointment**

Dear colleague, please provide your analysis regarding brief syncope; o/sat 98%, pulse 90, BP 144/92 cor reg s- pulm slight wheeze; dd: arrhythmia.

| Reason for referral | See above |
| --- | --- |
|  |  |
| Clinical notes | **Additional clinical information** |
|  |  |
|  | **Subconsultation** |
|  | (O) – Sys.BP:144  (O) – Diast.BP:92 |
|  |  |
|  | **Subconsultation** |
|  | (S) - briefly unconscious and, during this period of amnesia, collided with three cars and a bus. |
|  |  |
|  | **Subconsultation** |
|  | (S) – Voltaren gel quervain re |
|  |  |
|  | **Subconsultation** |
|  | (S) – pulse 98 |
|  |  |
|  | **Subconsultation** |
|  | (O)-Syst.BP:118  (O)-Diast.BP:74 |
|  |  |
| Relevant problem/episode list | **Problemlist**  Diabetes  Everything is okay  Myocardial infarction |
|  |  |
|  | **Episodelist**  Quervain re |
|  |  |
| Current medication | Acetylsalicylzuur tabl 80mg, 1.1T  Amitriptyline Tabl OMH 10mg, N 1T  Pantoprazol Tabl MSR 40mg, 1.1T  Atorvastatine Tabl OMH  Perindopril Tert-But T 8mg, 1.1T  Ivabradine Tabl OMH 5mg, 2.1T  Amlodipine Tabl 5mg, 1.1T  D-Cura Oral Solution 25000IE Amp, M11C  Nitroling Spr 0,4MG/D 250D, NITS |
|  |  |
| Contraindications related to medication | Ischemic heart disease (Incl. AP)  Hypertension  Diabetes without insuline |
|  |  |
|  |  |
| **Additional section** | Examination: systolic blood pressure 144 mmHg.  Examination: diastolic blood pressure 92 mmHg.  Examination: systolic blood pressure 118 mmHg.  Examination: diastolic blood pressure 74 mmHg. |

**Supplementary Table 1**

| **Differential Diagnoses (Phase-1)** | **Included Final diagnosis (Phase-3)** | **Calculation with penalty score 0.25*** | **DPS** |
| --- | --- | --- | --- |
| Cardiac syncope, Reflex syncope, PPS | Yes | +1 − (2 × 0.25) | 0.5 |
| Orthostatic hypotension, Reflex syncope | Yes | +1 − (1 × 0.25) | 0.75 |
| Cardiac syncope | No | 0 − (1 × 0.25) | −0.25 |
| Cardiac syncope, PPS, Orthostatic hypotension | No | 0 − (3 × 0.25) | −0.75 |
| Reflex syncope, Orthostatic hypotension, Cardiac syncope, PPS, Epilepsie | Yes | +1 − (4 × 0.25) | 0 |

***Penalty score see Methods**
